# Treatment of Diphtheria by Sub-Membranous Injections

**Published:** 1892-03-12

**Authors:** 


### TREATMENT OF DIPHTHERIA BY SUB-MEMBRANOUS INJECTIONS.

We recently discussed the latest views as to the nature of the diphtheritic microbe. The more definite results obtained in bacteriology have naturally led to new procedures in the local therapy of the disease. The mere removal of the false membrane is not advisable; that was attempted and abandoned many years ago as a useless waste of time and an unnecessary cause of pain to the patient.

Dr. A. Seibert, of New York, has reported thirty-five cases of pharyngeal diphtheria treated by submembranous injections. His instrument is practically a hypodermic syringe, having a long nozzle terminating in a hollow plate. At right angles to the latter are several very short ordinary hypodermic needles not over one-eighth of an inch long. The long nozzle can be bent on its axis at various angles, so as to fit the different portions of the throat. The fluid he employs for the injections is the *aqua chlori* of the United States Pharmacopœia. He bases his ideas upon the fact that the pharyngeal manifestations of diphtheria is, at the start, a local process, and that this owes its origin to the entry and penetration into the mucous membrane of the Klebs-Loeffler bacillus. He believes that the pseudo-membrane is not the disease, but the result of the disease, and is " a safe guide to the diphtheritic inflammation below it "; that the chief treatment should be local, and that the removal of pseudo-membranes is useless, as the bacilli contained therein are of no further consequence, and that local treatment, as carried out generally, does not reach the active bacilli in the lower strata of inflamed tissue, and is therefore, as a matter of fact, neither local nor germicidal. The wiping away of the pseudo-membranes and applying strong antiseptics to the parts is also ineffective, as only tending to cauterise and infect the healthy surrounding mucosa, to rubbing in the bacilli into deeper parts, and is without germicidal effect, therefore Professor Seibert has devised these instruments for the purpose of bringing comparatively small, but very strong, solutions into direct contact with the bacteria which are in activity upon the lower stratum of the mucosa. In using it the points are pressed firmly in to their full length into the pseudo-membrane, so as to reach the inflamed tissue below, and chlorine water is injected into the part. The chlorine is penetrating and attacks bacteria and all organic matter. It is better to inject deeply. He believes that where the membrane is of a dirty sloughing nature, there are more streptococci than bacilli present, and the observations of others lend support to his views on this point. The injections should be made on an empty stomach. The pain is slight. A mouth-wash is subsequently used of iodine and carbolic acid, and an ice-bag placed over the neck. Food and stimulants are given as usual. Professor Seibert found that the membrane remained for from two to four days, but it came away mechanically under the influence of the mouth-wash. He had lost only two cases out of the thirty-five, one from heart failure, and tracheo-bronchial diphtheria which had come on before he saw the case, and the other had been improperly treated by the attendants. Control cultures made in ten cases showed the Loeffler bacillus in each instance. Not one of the favourable cases had any subsequent paralysis, because the early germicidal action of the remedy prevented ptomaine formation.

How far we may safely assume that the results reported were due to this particular method we cannot pretend to say, but this new device is ingenious, and the results reported are distinctly in its favour.

M. Neveu, too, has practiced injections in the tissues of the tonsil, and in the largest and most accessible glands of the neck. Three to nine drops of a solution of distilled water, 90 grammes; finely powdered sublimate, 10 centigrammes (10 centigrammes morph.-hydrochlor. may be added) is used. M. Neveu also recommends complete antisepsis of the mouth and throat, and also application of tincture of iodine to the neck, larynx, and windpipe. For general treatment sulphate of quinine, naphthol, and salicylate of bismuth, and slight purgation are recommended.

We cannot help feeling that most practitioners will find great difficulty in conscientiously carrying out such methods of treatment, but it is well to be acquainted with the results observed by those who have overcome the difficulties of the situation.

